# Targeted NGS based hereditary autoinflammatory disorder screening in routine diagnostics, two year experience in the Netherlands

**DOI:** 10.1186/1546-0096-13-S1-P51

**Published:** 2015-09-28

**Authors:** MG Elferink, P van Zon, J Frenkel, W Harts, A Simon, A van Royen-Kerkhof, J Swart, H-K Ploos van Amstel, M van Gijn

**Affiliations:** 1 University Medical Center Utrecht, Medical Genetics, Utrecht, Netherlands; 2 University Medical Center Utrecht, Pediatric Immunology and Infectious diseases, Utrecht, Netherlands; 3 Radboud University Medical Center, Internal Medicine (NCIA), Nijmegen, Netherlands

## Introduction

Hereditary autoinflammatory diseases (AID) are characterized by recurrent bouts of systemic inflammation caused by dysregulation of the innate immunity system. The genotype-phenotype correlation can be highly variable which makes a genetic diagnosis in AID patients complex and laborious. A clear and definitive diagnosis cannot be provided for up to 80% of AID patients, which can be important for treatment options. To date, over 20 causal genes have been identified for monogenic AIDs.

## Patients and methods

We have developed a diagnostic method to facilitate genetic evaluation of the 23 known AID related genes at once using Next Generation Sequencing (NGS). We performed targeted *in-solution* enrichment followed by sequencing using the SOLID 5500 platform. An *in-house* developed bioinformatic pipeline was used to detect DNA variants in the selected AID genes. Cartagenia Bench lab NGS was used for filtering and classifying detected variants. Variants were confirmed by Sanger sequencing. Since May 2013, 120 patients have been tested with NGS.

## Results

We identified a genetic diagnosis in 17 patients (14%). Mutations were detected in classic autoinflammatory genes like *MVK*, *NLRP3* and *TNFRSF1A.* Moreover, we detected mutations in recently discovered genes, like *CECR1*, thereby demonstrating that regular updating of the targeted gene panel is warranted. Interestingly, we detected mosaicism for a pathogenic *NLRP3* mutation which had been missed by Sanger sequencing. Moreover in a patient with the phenotype of PAPA-syndrome, without a mutation in the PSTPIP1-gene, pathogenic compound heterozygous mutations in the MVK-gene were detected with NGS. This further supports the application of NGS based testing in these patients. In 20 additional patients (17%) only one disease causing allele was detected in genes involved in autosomal recessive disorders or the genetic results need further testing because the detected variants were of unknown clinical significance. The NGS method has been mainly applied to autoinflammatory patients other than FMF patients. In these patients diagnostic yield improved from 4% using Sanger sequencing single genes to 14% using the NGS based testing.

## Conclusions

Our results indicate that this NGS-based approach is an efficient strategy for rapid mutation detection in AID. Moreover, the increased diagnostic yield will give more insight in the genotype phenotype relationship for the different AID disorders enabling earlier diagnosis and better treatment.

